# Validation of PARX Models for Default Count Prediction

**DOI:** 10.3389/frai.2019.00009

**Published:** 2019-06-12

**Authors:** Arianna Agosto, Emanuela Raffinetti

**Affiliations:** ^1^Department of Economics and Management, University of Pavia, Pavia, Italy; ^2^Department of Economics, Management and Quantitative Methods, University of Milan, Milan, Italy

**Keywords:** credit risk, systemic risk, contagion, PARX models, validation measures

## Abstract

The growing importance of financial technology platforms, based on interconnectedness, makes necessary the development of credit risk measurement models that properly take contagion into account. Evaluating the predictive accuracy of these models is achieving increasing importance to safeguard investors and maintain financial stability. The aim of this paper is two-fold. On the one hand, we provide an application of Poisson autoregressive stochastic processes to default data with the aim of investigating credit contagion; on the other hand, focusing on the validation aspects, we assess the performance of these models in terms of predictive accuracy using both the standard metrics and a recently developed criterion, whose main advantage is being not dependent on the type of predicted variable. This new criterion, already validated on continuous and binary data, is extended also to the case of discrete data providing results which are coherent to those obtained with the classical predictive accuracy measures. To shed light on the usefulness of our approach, we apply Poisson autoregressive models with exogenous covariates (PARX) to the quarterly count of defaulted loans among Italian real estate and construction companies, comparing the performance of several specifications. We find that adding a contagion component leads to a decisive improvement in model accuracy with respect to the only autoregressive specification.

## 1. Introduction

The credit market is experiencing a large growth of innovative financial technologies (fintechs). In particular, peer-to-peer lending platforms propose a business model that disintermediates the links between borrowers and lenders and is based on a stronger interconnectedness between the agents with respect to the traditional banking system. Furthermore, peer-to-peer lenders often do not have access to individual borrowers' data usually employed in banks' credit scoring models, such as financial ratios and credit bureau information. In this context, models analyzing correlation in the default dynamics of different agents or sectors can effectively support credit risk assessment.

More generally, interconnectedness, already known as a trigger of the great financial crisis in 2008–2009, is recognized as a source of *systemic risk*, i.e., according to the European Central Bank, “the risk of experiencing a strong systemic event, which adversely affects a number of systemically important intermediaries or markets.” The impact that an event experienced by an economic agent or sector can have on other institutions in the market is often referred to as *contagion*. From an econometric viewpoint, statistical methods able to properly measure the systemic risk that arises from interconnectedness are necessary to safeguard both traditional intermediaries and peer-to peer lending investors, therefore maintaining financial stability.

The first systemic risk measures have been proposed for the financial sector, in particular by Adrian and Brunnermeier (2016) and Acharya et al. (2012). These works consider financial market data, calculating the estimated loss probability distribution of a financial institution, conditional on an extreme event in the financial market. Being applied to market prices, these models are based on Gaussian processes.

Financial market data have also been used in another recent approach to systemic risk, based on correlation network models, where contagion effects are estimated from the dependence structure among market prices. The first contributions in this framework are Billio et al. (2012) and Diebold and Yilmaz (2014), who derived contagion measures based on Granger-causality tests and variance decompositions. Ahelegbey et al. (2016) and Giudici and Spelta (2016) have extended the methodology introducing stochastic correlation networks.

Networks represent a relevant modeling approach in peer-to-peer platforms, where continuous credit demand and lending activity makes available large amounts of transaction data. Network models have been recently applied to peer-to-peer lending platforms data by Ahelegbey et al. (2019) and Giudici et al. (2019).

Another possible approach to analyze contagion is to build discrete data models for the counts of default events. Including exogenous covariates in such models allows to test whether the failure of a given firm increases the probability that other failures occur conditional on a set of risk factors. For example, Lando and Nielsen (2010) model default times by Poisson processes with macroeconomic and firm-specific covariates entering the default intensities. Their methodology does not directly include a contagion component, but investigates possible contagion effects by testing whether the Poisson model is misspecified. Default counts are also modeled by Koopman et al. (2012) and, recently, by Azizpour et al. (2018), who use a binomial specification where the probability of default is a time-varying function of underlying factors, also including unobserved components.

Among the approaches to default counts modeling we focus on PARX models developed by Agosto et al. (2016), including autoregressive and exogenous effects in a time-varying Poisson intensity specification. A recent extension by Agosto and Giudici (Submitted) makes PARX models suitable to investigate default contagion. In this paper, PARX models are applied to default counts data in the Italian real estate sector.

Validation is a critical issue in credit risk modeling, because of the interest in selecting indicators able to predict the default peaks, and achieves further importance in artificial intelligence systems, where the traditional accuracy measures based on probabilistic assumptions cannot always be implemented.

In the specific case of contagion analysis, such as the one presented in this paper, model selection also assumes an explanatory role: the comparison of alternative specifications, including contagion components or not and considering different exogenous risk factors, provides a deeper insight into default correlation.

In our empirical application we validate the models applied to default counts using several measures, including the Rank Graduation index *RG*, recently developed by Giudici and Raffinetti (Submitted). In Giudici and Raffinetti (Submitted), the purpose was to propose an index that is objective and not endogenous to the system itself. The Rank Graduation index (*RG*) was originally developed to deal with two real machine learning applications characterized, respectively, by a binary and a continuous response variable. It is based on the calculation of the cumulative values of the response variable, re-ordered according to the ranks of the values predicted by the considered model. Giudici and Raffinetti (Submitted) showed that the *RG* metric is more effective than the *AUROC* (typically used for models with binary response variables) and the *RMSE* (typically used for models with continuous response variables). Specifically, in the binary case, it appears as an objective predictive accuracy diagnostic, since built on re-ordering the response variable values according to the predicted values themselves, and, in the continuous case, it is not affected by the presence of outliers. Here, the application of the Rank Graduation index is extended to the case of default count data and the related results are compared to those obtained with traditional measures, such as the likelihood-based criteria and *RMSE*. Given its attractive features and properties, both regulators and supervisors may be interested in the *RG* employment in artificial intelligence applications, in order to better understand and manage the business models and avoid decisions based upon wrong outputs which may lead to losses or reputational risks.

The paper is organized as follows. Section 2 describes PARX models and how they can be used to study the default count dynamics and investigate possible contagion effects. Section 3 provides an overview of the main validation criteria and the basic elements characterizing the Rank Graduation measure. Section 4 presents the empirical findings derived from the application and validation of PARX models for default counts. Section 5 concludes.

## 2. PARX Models

The approach to default counts modeling applied in this work is based on PARX models (Agosto et al., 2016). PARX models assume that a count time series *y*_*t*_, conditional on its past, follows a Poisson distribution with a time-varying intensity λ_*t*_ > 0, whose formulation includes an autoregressive part and a *d*-dimensional vector of exogenous covariates $x_{t}:={\left(x_{1t},x_{2t},\ldots ,x_{dt}\right)}^{\prime }\in {\mathbb{R}}^{d}$:

(1) $$\begin{array}{r} y_{t}|F_{t-1}\sim Poisson\left(\lambda_{t}\right)\Leftrightarrow P\left(y_{t}=y|F_{t-1}\right)=\frac{\lambda_{t}^{y}\exp\left(-\lambda_{t}\right)}{y!}\\ \lambda_{t}=\omega +\overset{p}{\underset{i=1}{\sum }}\alpha_{i}y_{t-i}+\overset{q}{\underset{i=1}{\sum }}\beta_{i}\lambda_{t-i}+\overset{d}{\underset{i=1}{\sum }}\gamma_{i}f\left(x_{i}\right) \end{array}$$

with $F_{t-1}$ denoting the σ-field σ{*y*_0_, …, *y*_*t*−1_, λ_0_, …, λ_*t*−1_, *x*_0_, …, *x*_*t*−1_}, ω > 0, α_*i*_ ≥ 0 (*i* = 1, 2, …, *p*) and *β*_*i*_ ≥ 0 (*i* = 1, 2, …, *q*).

When the vector of unknown parameters *γ*: = (*γ*_1_, …, *γ*_*d*_) is null, the model reduces to Poisson Autoregression (PAR) developed by Fokianos et al. (2009), who showed how including past values of the intensity λ_*t*_ allows for parsimonious modeling of long memory effects. Note that exogenous covariates are included through a non-negative link function to guarantee that intensity is positive.

The presence of both dynamic and exogenous effects makes PARX models suitable for describing count time series of events that cluster in time, as defaults are known to do. Furthermore, it can be shown that including an autoregressive component as well as covariates in a Poisson process generates overdispersion, that is unconditional variance larger than the mean, a typical feature of default count time series.

Agosto et al. (2016) applied model (1) to Moody's rated US corporate default counts, with the aim of distinguishing between the impact of past defaults on current default intensity—possibly due to contagion effects—and the impact of macroeconomic and financial variables acting as common risk factors. Recently, Agosto and Giudici (Submitted) proposed to extend PARX models to accomplish investigation of default contagion effects. Differently from model (1) and following Fokianos and Tjøstheim (2011), they use a log-linear intensity specification. This allows to consider negative dependence on exogenous covariates, which can be useful in credit risk applications.

Letting *y*_*jt*_ the number of defaults in economic sector (or, more generally, group of borrowers) *j* at time *t* and *y*_*kt*_ the number of defaults in sector *k*, they define the following model:

(2) $$\begin{array}{l} y_{jt}|F_{t-1}\sim Poisson(\lambda_{jt})\\ \log(\lambda_{jt})=\omega_{j}+\sum_{i=1}^{p}\alpha_{ji}\log(1+y_{jt-i})+\sum_{i=1}^{q}\beta_{ji}\log(\lambda_{jt-i})\\ +\sum_{i=1}^{r}\gamma_{ji}x_{t-i}+\sum_{i=1}^{s}\zeta_{ji}\log(1+y_{kt-i}) \end{array}$$

with ω_*j*_, α_*ji*_, β_*ji*_, γ_*ji*_, ζ_*ji*_ ∈ ℝ and $x_{t-i}:={\left(x_{1t-i},x_{2_{t}-i},\ldots ,x_{dt-i}\right)}^{\prime }\in {\mathbb{R}}^{d}$ being a vector of lagged exogenous covariates. In model (2), that the authors call Contagion PARX, ζ_*j*_ measures the effect of the covariate default count process on the response default counts, which can be interpreted as a contagion effect. Taking the log(·) + 1 of counts allows to deal with possible zero values. This specification can easily be extended to the case where the default counts of a set of different sectors, rather than only one covariate default series, are included among the regressors.

## 3. Model Validation

A basic issue of the artificial intelligence systems is the validation process for the model prediction quality assessment. In this paper, we consider the available literature for validation procedures and illustrate a new practice for the validation.

In literature, several metrics aimed at comparing and improving the models are available, depending on the nature of data. As mentioned above, one of the focus of this paper is on the use of the Poisson autoregressive models for modeling default counts. The presence of a discrete response variable suggests the choice of the Root Mean Squared Error (RMSE) and the criteria based on likelihood, such as the Akaike Information Criterion (AIC) and Bayesian Information Criterion (BIC), as the most widely employed measures for the model predictive accuracy evaluation.

It is worth noting that in the model validation research field, the lack of a standard metric, working regardless of the nature of the response variable to be predicted, is still a crucial drawback to be faced. Recently, Giudici and Raffinetti (Submitted) have worked out one possible solution by proposing a new measure, the *RG* Rank Graduation index, which is based on the calculation of the cumulative values of the response variable, according to the ranks of the values predicted by a given model. The main features of the *RG* criterion together with a brief description of the conventional validation measures are provided in the following subsections.

### 3.1. Conventional Model Validation Measures

The RMSE, AIC, and BIC criteria, intended as some of the most broadly used metrics for the model validation, are defined as follows:

(3) $$\begin{array}{r} RMSE=\sqrt{\frac{1}{\text{n}}\sum_{i=1}^{n}{\left({\hat{y}}_{i}-y_{i}\right)}^{2}}, \end{array}$$

where the *y*_*i*_'s and ŷ_*i*_'s represent the response variable observed and predicted values (with *i* = 1, …, *n*), respectively,

(4) $$\begin{array}{r} AIC=-2logL\left(\hat{\theta }|x_{1},\ldots ,x_{n}\right)+2k \end{array}$$

and

(5) $$\begin{array}{r} BIC=-2logL\left(\hat{\theta }|x_{1},\ldots ,x_{n}\right)+klog\left(n\right), \end{array}$$

where θ is the set of model parameters, $logL\left(\hat{\theta }|x_{1},\ldots ,x_{n}\right)$ is the log-likelihood of the model given the data *x*_1_, …, *x*_*n*_ when evaluated at the maximum log-likelihood estimate of θ $\left(\hat{\theta }\right)$, *k* is the number of the estimated parameters in the model and *n* is the number of observations.

The best model, in terms of predictive accuracy, is the one that provides the minimum RMSE, AIC and BIC (for more details, see e.g., Kuha, 2004; Hyndman and Koehler, 2006).

### 3.2. The *RG* as an Additional Model Validation Criterion

Besides the conventional model validation criteria, the *RG* measure deserves a wider discussion, especially because it appears as a more general predictive accuracy criterion which does not depend on the type of data to be analysed. As mentioned above, in Giudici and Raffinetti (Submitted), the *RG* was proposed as a unique metric to assess the model predictive accuracy in presence of both binary and continuous response variables. Moreover, due to its features and construction it fulfills some attractive properties: (1) it appears as an objective criterion compared with the *AUROC* metric, which depends on the arbitrary choice of the cut-off points; (2) it is a robust criterion since non-sensitive to the presence of outliers. Given the topic of this paper, related to the employment of discrete data models for default counts, it is therefore worth to extend the frontiers of the *RG* application areas to the context of discrete response variables.

The interest in applying the *RG* index to default count data is also linked to some typical features shown by the time series of defaults. The common presence of peaks and outliers makes indeed preferable to evaluate predictive accuracy of default count models through concordance measures rather than error measures that are known to be sensitive to outliers.

In order to better highlight the main strengths of our validation approach, a brief overview on the *RG* construction seems to be basic. The proposal is based on the so-called *C* concordance curve, which is obtained by ordering the normalized *Y* response variable observed values according to the ranks of the predicted Ŷ values provided by the model.

**Figure 1:**
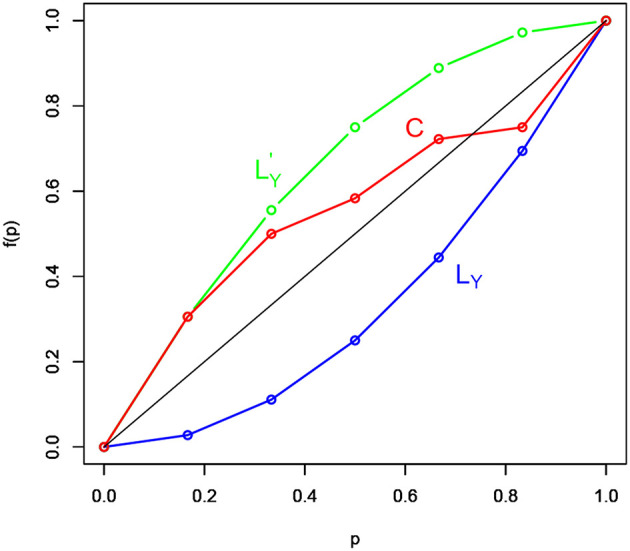
The *L*_*Y*_ (blue) Lorenz curve, dual $L\prime_{Y}$ (green) Lorenz curve, and the *C* (red) concordance curve.

Let *Y* be a discrete response variable and let *X*_1_, …, *X*_*p*_ be a set of *p* explanatory variables. Suppose to apply a model such that ŷ = *f*(**X**). The model predictive accuracy is assessed by measuring the distance between the set of the *C* concordance curve points, whose coordinates are denoted with $\left(i/n,\left(1/\left(nȳ\right)\right)\overset{i}{\underset{j=1}{\sum }}y_{{\hat{r}}_{j}}\right)$, where $ȳ=\frac{1}{n}\overset{n}{\underset{i=1}{\sum }}y_{i}$ and $y_{{\hat{r}}_{j}}$ represents the *j*-*th* response variable value ordered by the rank of the corresponding predicted value ŷ_*j*_ (with *j* = 1, …, *i* and *i* = 1, …, *n*), and the set of the bisector curve points of coordinates (*i*/*n, i*/*n*). As an example, the graphical representation of the *C* concordance (in red) and bisector (in black) curves is displayed in Figure 1. Figure 1 reports also two other curves: the response variable *L*_*Y*_ Lorenz curve (in blue), which is defined by the normalized *Y* values ordered in non-decreasing sense, and the response variable $L\prime_{Y}$ dual Lorenz curve (in green), which is defined by the normalized *Y* values ordered in non-increasing sense.

Both the response variable Lorenz and dual Lorenz curves take a remarkable role in the *RG* measure construction, especially the response variable *L*_*Y*_ Lorenz curve. Indeed, since the model predictive accuracy degree depends on the distance between the bisector and the *C* concordance curves, it follows that the more the *C* concordance curve moves away from the bisector curve, the more the model predictive accuracy improves. This because the bisector curve detects a model without predictive capability. Indeed, if ŷ_*i*_ = ȳ, for any *i* = 1, …, *n*, through some manipulations, the coordinates of the *C* concordance curve becomes (*i*/*n, i*/*n*), which perfectly corresponds to the coordinates of points characterizing the bisector curve. Analogously, if the *C* concordance curve perfectly overlaps with the *L*_*Y*_ Lorenz curve, then the model is perfect because it preserves the ordering between the observed response variable *Y* values and the corresponding Ŷ estimated values. In such a case, the coordinates of the *C* concordance curve become $\left(i/n,\left(1/\left(nȳ\right)\right)\overset{i}{\underset{j=1}{\sum }}y_{\left(j\right)}\right)$, where *y*_(*j*)_'s, with *j* = 1, …, *i* and *i* = 1, …, *n*, are the response variable values ordered in non-decreasing sense.

Based on the above considerations, the *RG* measure takes the following expression:

(6) $$\begin{array}{r} RG=\sum_{i=1}^{n}\frac{{\left\{(1/(n\bar{y}))\sum_{j=1}^{i}y_{{\hat{r}}_{j}}-i/n\right\}}^{2}}{i/n}=\sum_{i=1}^{n}\frac{{\left\{C(y_{{\hat{r}}_{i}})-i/n\right\}}^{2}}{i/n}, \end{array}$$

where $C\left(y_{{\hat{r}}_{j}}\right)=\frac{\overset{i}{\underset{j=1}{\sum }}y_{{\hat{r}}_{j}}}{\overset{n}{\underset{i=1}{\sum }}y_{i}}$ represents the cumulative values of the (normalized) response variable *Y*. The *RG* measure in (6) appears as an absolute metric, since it takes values in the close range [0, *RG*_*max*_], where *RG*_*max*_ is the maximum value that can be achieved. Trivially, the maximum *RG* value can be reached if the model perfectly explains the response variable, meaning that the *C* concordance curve indifferently overlaps with the response variable Lorenz or dual Lorenz curves. Indeed, the distance between the *Y* Lorenz or dual Lorenz curves and the bisector curve is the same, being the two curves symmetric around the bisector curve. A normalized *RG* measure is then defined as the ratio between the absolute *RG* measure ad its maximum value *RG*_*max*_.

Finally, we remark that when some of the Ŷ values are equal to each other, we take into account the adjustment suggested by Ferrari and Raffinetti (2015) in order to solve the re-ordering problem. Specifically, the original *Y* values associated with the equal Ŷ values are substituted by their mean.

## 4. Application

In this section we provide the application of PARX models to Italian corporate default counts data in the real estate sector and their evaluation through different validation measures. Bank of Italy's Credit Register collects the quarterly number of transitions to *bad loans* in major economic sectors. Bad loans are exposures to insolvent debtors that cannot be recovered and that the bank must report as balance sheet losses. Being an absorbent state, the number of loans turned out to be *bad* in a given period can be used as a proxy of the default count at that time. The data are quarterly and divided by economic sector. Among the sectors included in the database we focus on the Real Estate and Commercial ones, using data covering the period March 1996–June 2018 (90 observations). The real estate sector includes both real estate and construction companies and was one of the most hit by the recent financial crisis. Our choice is motivated by the economic interest in verifying the impact that the default dynamics of commercial firms, highly influenced by the changes in consumption behavior, may have on the real estate sector. Possible contagion from the commercial to the real estate sector is mainly due to the decrease of both business and private investments by the owners of commercial activities, causing a reduction in the demand of new buildings and real estate services.

**Figure 2:**
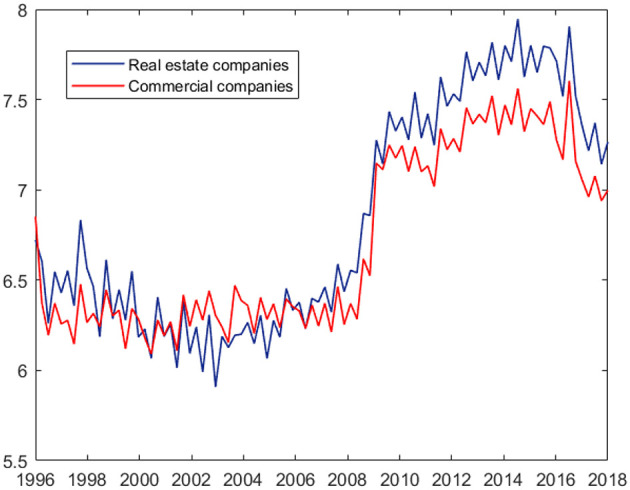
Default count time series of real estate and commercial corporate sectors (logarithmic scale): Italian data.

**Figure 3:**
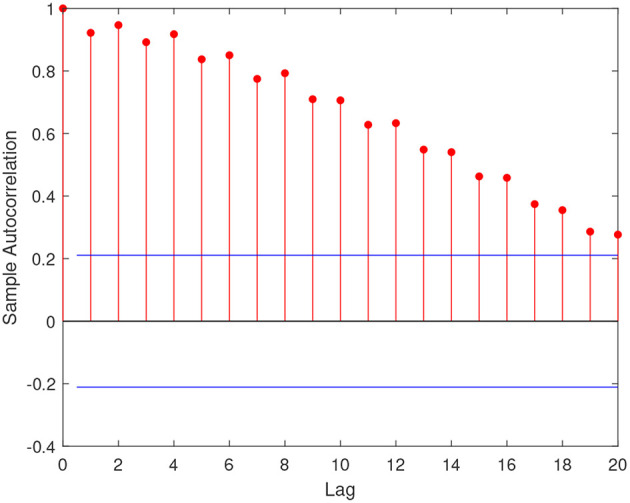
Sample autocorrelation function of real estate default count time series: Italian data.

Figure 2 shows the default count time series of the two economic sectors considered. Both series exhibit clustering and a possible structural break in 2009, with an increase in both level and variability. Table 1 reports the main summary statistics for the response variable of our exercise, that is the default counts among real estate Italian firms, while Figure 3 shows the autocorrelation function of the series. Both the presence of overdispersion (the empirical variance is 506468.7 and the empirical average 1132.9) and the slowly decaying autocorrelation encourage the use of PARX to model the data.

**Table 1:** Summary statistics for the real estate sector default counts: Italian data.

**Mean**	**Std. Dev**.	**Min**	**Max**
1132.9	711.7	368	2825

To investigate credit contagion effects between the two sectors and show our validation procedure, we consider the model regressing real estate sector default counts on their past values and on past commercial sector default counts.

An important robustness and validation step when applying PARX models is assessing the effects of including exogenous covariates summarizing the macroeconomic context, such as the business cycle. The aim is to verify to what extent the macroeconomic stress affecting all the economic agents and sectors explains the default and contagion dynamics.

Thus, we first estimate a model (Full Contagion PARX) that, according to specification (2), includes both a contagion component and the exogenous covariate GDP in a log-linear intensity specification^1^:

(7) $$\begin{array}{l} \log(\lambda_{t})=\omega +\alpha \log(1+y_{t-1})+\gamma_{1}GDP_{t-1}+\gamma_{2}GDP_{t-2}\\ \;+\zeta_{1}\log(1+y_{Ct-1})+\zeta_{2}\log(1+y_{Ct-2}) \end{array}$$

where *GDP*_*t*_ is the Italian GDP growth rate and *y*_*Ct*_ is the number of defaults among commercial sector companies at time *t*.

From Table 2, reporting the parameter estimates for the model above, note that the effect of GDP variation on the real estate sector default risk is significant at the second lag, suggesting a delayed effect of the business cycle on the corporate solvency dynamics which is reasonable from an economic point of view. Also the impact of commercial sector default counts turns out to be significant with a two quarters lag.

**Table 2:** Parameter estimates for real estate sector default counts.

**Variable**	**Estimate**	**Standard error**	***t*-stat**
Constant	−0.1339	0.3285	0.4075
Real estate sector bad loans in t-1	0.6062	0.1591	3.8103^***^
Commercial sector bad loans in t-1	−0.2886	0.2689	−1.0732
Commercial sector bad loans in t-2	0.7161	0.1299	5.5129^***^
GDP growth rate in t-1	−0.0341	0.0284	−1.2009
GDP growth rate in t-2	−0.0705	0.0274	−2.5732^**^

*** *p < 0.001*;

** *p < 0.01*.

In order to highlight the contribution of the different components—autoregressive, contagion, and exogenous—and validate the model we then consider two alternative specifications.

We first estimate a PARX model that, following specification (1), includes an autoregressive and an exogenous component in a linear intensity specification:

(8) $$\begin{array}{r} \lambda_{t}=\omega +\alpha y_{t-1}+\gamma_{1}GDP_{t-1}^{-}+\gamma_{2}GDP_{t-2}^{-} \end{array}$$

where $GDP^{-}:={𝕀}_{GDP<0}|GDP|$, that is the absolute value of the negative part of GDP growth rate. This ensures that default intensity is positive, as needed in the linear specification. Fitting the model above, we do not find significant effects of GDP decrease on the real estate sector. Thus, the model reduces to an only autoregressive Poisson model as the previously cited PAR. According to this result, while negative correlation with the business cycle taken into account by the log-linear model significantly explains the default dynamics, the positive association between the GDP decrease and the default counts is not significant in our exercise. This highlights the advantage of using specifications that allow to consider negative dependence.

The last competing model is a Contagion PARX without other covariates than commercial sector default counts [γ parameters equal to 0 in specification (2)]:

(9) $$\begin{array}{r} \log\left(\lambda_{t}\right)=\omega +\alpha y_{t-1}+\zeta_{1}\log\left(1+y_{Ct-1}\right)+\zeta_{2}\log\left(1+y_{Ct-2}\right) \end{array}$$

We now compare the in-sample performances of the three models above: PAR model, Contagion PARX model, and Full Contagion PARX model by using the RMSE, AIC, BIC and *RG* validation measures. The results are illustrated in Table 3.

**Table 3:** Validation measures for the considered models.

**Model**	**RMSE**	**AIC**	**BIC**	***RG***
Full contagion PARX model	207.68	−1,256,019	−1,256,004	6.098
Contagion PARX model	222.02	−1,255,643	−1,255,633	6.114
PAR model	272.06	−1,254,332	−1,254,327	5.796

First note that the Full Contagion PARX model is the most performing according to RMSE, AIC, and BIC criteria. In particular, moving from the PAR to the Contagion PARX specification leads to a decrease of nearly 24% in the RMSE. The model ordering changes when considering the *RG* index. The model showing the higher *RG* index is indeed the Contagion PARX one, with a value of 6.114. The Full Contagion PARX model shows a slightly lower value (6.098), while the *RG* index of the PAR model is 5.796. As *RG*_*max*_ = 6.709, it follows that the PAR model explains the 86.4% of the variable ordering, compared with the 90.9% of the Full Contagion PARX Model and the 91.1% of the Contagion PARX Model.

According to all the considered measures, adding the contagion component leads to a decisive increase in model performance with respect to the only autoregressive specification, with a decrease of 18% in RMSE and an increase of nearly 3.5% in accuracy. Considering the negative association between the macroeconomic stress and default risk considerably reduces the error measure—the decrease in RMSE with respect to the Contagion PARX model is around 7% - but does not improve model performance in terms of accuracy, measured through the *RG* index. In such a case, the choice of the preferable specification depends on the objective of model comparison. If the aim, as in our contagion analysis, is validating a model that well explains the empirical distribution of the data even with a limited number of parameters, rather than getting a point forecast of the response variable, decisions based on a concordance measure are more appropriate.

## 5. Conclusion

In this paper, we have illustrated an application of PARX models, which investigate contagion through Poisson autoregressive stochastic processes, and we have evaluated the predictive accuracy of different specifications. While previous works focused on the theory development and extension of PARX, we concentrate on the issue of validating these models and measuring the contribution of contagion and exogenous components to their predictive performance. For doing so, we resorted to a novel metric, called *RG* index, which is independent on the involved response variable nature. Specifically, the *RG* measure, originally considered in the cases of binary and continuous data, was here extended with the aim of covering also the case of discrete data.

Fitting several PARX-type specification to the quarterly count of defaulted loans in the Italian real estate sector, we find evidence of a significant effect of commercial sector defaults on real estate default risk. We also find that considering the effect of the business cycle improves model performance according to likelihood-based criteria and traditional error measures, but it does not increase predictive accuracy according to the new concordance metric.

## Data Availability

Publicly available datasets were analyzed in this study. This data can be found here: https://www.bancaditalia.it/statistiche/basi-dati/bds/index.html.

## Author Contributions

AA is a post-doctoral research fellow at University of Pavia, Department of Economics and Management. She has a research experience in Econometrics and Quantitative Finance with application to risk management, especially credit risk and contagion models. ER is Assistant Professor in Statistics at University of Milan, Department of Economics, Management, and Quantitative Methods. She has a research experience in dependence analysis and model validation criteria.

### Conflict of Interest Statement

The authors declare that the research was conducted in the absence of any commercial or financial relationships that could be construed as a potential conflict of interest.

## Footnotes

^1^The number of lags has been determined through preliminary model selection based on likelihood ratio and BIC criterion.
